# Hybrid Thermoplastic Composites from Basalt- and Kevlar-Woven Fabrics: Comparative Analysis of Mechanical and Thermomechanical Performance

**DOI:** 10.3390/polym15071744

**Published:** 2023-03-31

**Authors:** Hafsa Jamshaid, Rajesh Kumar Mishra, Vijay Chandan, Shabnam Nazari, Muhammad Shoaib, Laurent Bizet, Petr Jirku, Miroslav Muller, Rostislav Choteborsky

**Affiliations:** 1 School of Engineering and Technology, National Textile University, Faisalabad 37610, Pakistan; 2 Department of Material Science and Manufacturing Technology, Faculty of Engineering, Czech University of Life Sciences Prague, Kamycka 129, 165 00 Prague, Czech Republic; 3 Department of Sustainable Technologies, Faculty of Tropical Agriscience, Czech University of Life Sciences Prague, Kamycka 129, 165 00 Prague, Czech Republic; 4 Department of Mechanical and Production Engineering, Normandie Université, UNIHAVRE, LOMC, CNRS UMR 6294, 76610 Le Havre, France

**Keywords:** basalt, Kevlar, hybrid composite, thermoplastic, mechanical test, thermogravimetric analysis (TGA), differential scanning calorimetry (DSC), scanning electron microscopy (SEM)

## Abstract

Current research deals with thermoplastic polyamide (PA6)-based composites reinforced with basalt and Kevlar fabrics. Hybrid composites were developed by altering the stacking sequence of basalt and two kinds of Kevlar fabrics. Pure-basalt- and pure-Kevlar-based samples were also developed for comparison purposes. The developed samples were evaluated with respect to mechanical and thermomechanical properties. Mechanical tests, e.g., tensile, flexural, and impact strength, were conducted along with thermogravimetric analysis (TGA) and differential scanning calorimetry (DSC) to ascertain the load-bearing and high-temperature stability of the hybrid composite samples vis-à-vis pure-basalt- and Kevlar-based samples. Scanning electron microscopy (SEM) was carried out to study the nature of fracture and failure of the composite samples. The pure-basalt-based PA6 thermoplastic composites exhibited the best mechanical performance. Hybridization with basalt proved to be beneficial for improving the mechanical performance of the composites using Kevlar fabrics. However, a proper stacking sequence and density of Kevlar fabric has to be selected. The thermogravimetric analysis showed minimal weight loss for basalt-based composites. Furthermore, the thermal stability of the composites using Kevlar fabric was improved by hybridization with basalt fabric. The thermomechanical characteristics of hybrid composites may be altered by changing the stacking order of the reinforcements. Differential scanning calorimetry further established that the hybrid composites with alternate layers of basalt and Kevlar can improve the heat flow rate and enable survivability at extreme temperatures. Such novel hybrid composites can be used for high-load-bearing and high-temperature applications, e.g., defense, aerospace, automotives, and energy applications.

## 1. Introduction

Conventional materials such as metals and ceramics are experiencing reduced importance due to their corrosive nature, high weight, and high maintenance cost [1]. Polymer composite materials are used as alternatives to conventional materials due to their superior properties [2,3,4,5]. Fiber-reinforced polymer composites (FRPs) have seized the attention of researchers and are used in automotive, marine, aerospace, defense, and sports with engineering properties [6,7,8,9]. Having distinct features such as high strength-to-weight ratio, toughness, rigidity, thermal resistance, energy absorption capability, low density, and high stiffness are the major advantages of such materials over conventional materials [10,11]. In applications, they may be put through different loading conditions of mechanical and thermal stress. With the passage of time and with loading, there is a loss in strength due to fiber breakage, and delamination in the FRPs [12,13]. These are high-performance textiles and several cutting-edge research studies have been reported about applications in modern society [14,15,16,17,18,19].

The performance of the composites can be enhanced by the hybridization of different fibers in the same matrix (hybrid composites) as the remedy for such problems [20,21,22,23,24]. Several kinds of research studies about hybrid composites such as carbon/glass, Kevlar/glass, Kevlar/carbon, carbon/flax, polylactic acid (PLA)/hemp, carbon/aramid, jute/glass, and jute/carbon fibers have been reported in literature [25]. All these thermoset composites are examined through mechanical characterizations. Kevlar fiber has popularity in making FRP composites due to its excellent properties. Kevlar-reinforced composites offer advanced properties due to high-energy absorbance, high tensile strength, and high tensile-to-compression-strength ratio [26]. Moreover, it has a lower compressive strength as compared to glass fiber and carbon fibers. Therefore, Kevlar-reinforced composites (KRCs) tend to fail easily under compressive loadings. It can improve compressive strength by hybridizing with other fibers, e.g., glass and carbon [27,28,29,30,31,32,33].

The hybridization of fibers is an effective technique to improve the properties of Kevlar-reinforced composites, allowing the manufacturer to tailor the properties according to the needs [34]. Recently, basalt fibers gained more importance over glass and carbon fibers due to superior properties, being naturally abundant, and being environmentally friendly [35]. Basalt and Kevlar work synergistically in terms of their properties. Kevlar shows a lower compressive strength and is weaker in the transverse direction; hence, a basalt/Kevlar hybrid composite is one of the best options for FRP composites with improved mechanical properties. It is cheaper in price and offers eco-friendly options [36]. From the literature, it can be seen that the impact response is improved by the combination of fibers such as Kevlar with basalt and glass [37]. Basalt/nylon with varying percentages of nylon showed a significant improvement in impact properties [38]. Carbon/basalt hybrid composites with various impact energies showed that alternate layers of fibers resulted in high-energy absorption [39]. In the case of 3-D woven Kevlar/basalt epoxy composites, there was high-energy absorption in interply laminate as compared to intraply energy absorption [40]. In 3D woven angle-interlock Kevlar/basalt-reinforced polypropylene hybrid composites, superior properties were reported as compared to the individual ones [41]. In carbon/basalt composites, a positive hybridization effect can be seen in all stacking sequences [42].

High-performance composites are mostly obtained from thermoset resins that take a longer time for manufacturing and curing. Such composites are difficult to recycle due to the presence of catalyst/hardener along with resin. On the other hand, thermoplastic composites have numerous advantages, e.g., better impact properties, ductility, environmental resistance, and recyclability. However, the problem is their high viscosity, which makes the impregnation of reinforcement difficult, especially when the fiber volume fraction (Vf) is higher. This results in prolonged impregnation time and sometimes poorly impregnated areas, which leads to mechanical failures. To overcome these issues, reactive thermoplastic resin such as PA6 was developed [43,44,45,46,47]. Several researchers have reported the mechanical advantages of thermoplastic composites produced by pultrusion and reinforced with fibrous materials [48,49,50,51].

The novelty of the study is the hybridization of woven basalt and Kevlar fabrics as a reinforcement in thermoplastic composites. The current research aimed to investigate the benefits of fiber hybridization with high-fluidity polyamide-6 thermoplastic resin using different layering/stacking sequences. Individual reinforcement layers of basalt- and Kevlar-woven fabrics were also investigated for comparison. Effects of fabric characteristics and hybridization were evaluated by visual observation, optical microscopy, mechanical performance tests such as tensile, flexural, and impact strength, as well as thermal characterizations such as Thermogravimetric Analysis (TGA) and Differential Scanning Calorimetry (DSC). These novel hybrid composites can be used for high-performance applications such as sports, automotive, and aerospace.

## 2. Materials and Methods

### 2.1. Materials

High-performance fibers, e.g., basalt (Supplied by Kamenny Vek, Dubna, Russia) and Kevlar (Supplied by Dupont, Praha, Czech Republic), were used for the present research. Plain woven basalt and Kevlar fabrics were developed by weaving in the National Textile University, Faisalabad. All the fabrics were produced on a CCI sample weaving machine (CCI Tech, Taipei, Taiwan). Specifications of the reinforcement fabrics are given in Table 1.

For achieving a constant fiber volume fraction (Vf) of 45% (0.45) and thickness of 1.1 mm in all composite samples, different plies were used for basalt and Kevlar fabrics. Thermoplastic resin PA6 of brand name Evolite™ XS1480 of density 1.13 g/cm^3^ was supplied by Solvay Chemicals (Brussels, Belgium) in the form of powder (black). Its properties are given in Table 2.

#### Fourier Tranform Infrared (FTIR) Analysis (ATR Mode) and Energy-Dispersive X-ray (EDX) of the Basalt Fabric

Attenuated total reflection (ATR) Fourier transform infrared (FTIR) spectroscopy (NicoletiZ10, Thermo Fisher Scientific Corporation, Pardubice, Czech Republic) was used in order to understand the chemical structure of the fiber. The transmission method was used with 16 scans for a background and 16 scans for a sample, and the spectral range was 4000–7500 cm^−1^ with a resolution of 4/cm.

Elemental detection via electron dot-mapping was conducted using energy-dispersive X-ray (EDX) analysis on the scanning electron microscope MIRA 3 TESCAN (Brno, Czech Republic) for basalt fiber to ascertain the chemical composition.

**Figure 1:**
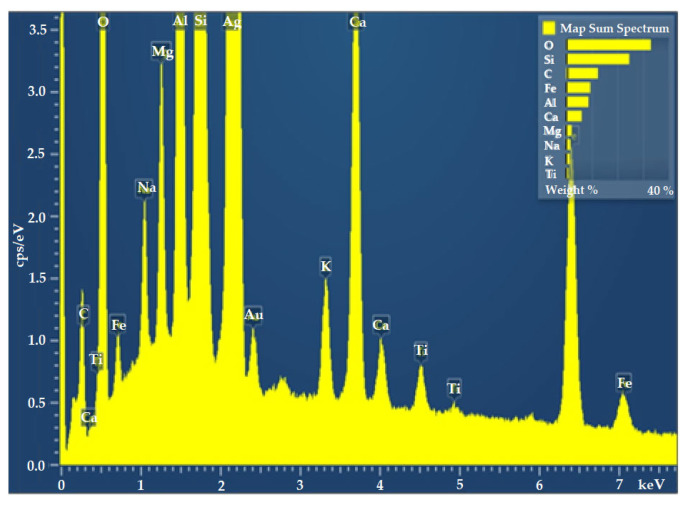
Energy-dispersive FTIR analysis of basalt.

Table 3 and Figure 1 show the summary of elements detected in energy-dispersive X-ray (EDX) analysis. Spectral analysis detected a number of elements in basalt. The presence of silica (Si) (24.58 wt%) and oxygen (O) (32.98 wt%) was found to be dominant as compared to other elements. 

From a chemical composition point of view, SiO_2_ and Al_2_O were the dominant compounds. The content of FeO and Fe_2_O_3_ plays a very important role in determining many physicomechanical properties of basalt fibers, such as density, color (from brown to dull green, depending on the FeO content), lower heat conduction, and better temperature stability compared to E-glass fibers. The chemical composition of basalt rocks influences the properties of the resulting fibers, fabrics, and composites. 

Basalt can be suited for fire protective applications and so it can replace almost all applications of asbestos, which poses health hazards by damaging respiratory systems as fiber should have a diameter above 5 µm and have sufficient length.

Basalt fibers and fabrics are labeled as safe according to both the USA and the European occupational safety guidelines. Its particles or fibrous fragments due to abrasion are too thick to be inhaled and deposited in the lungs, but care in handling is recommended.

### 2.2. Methods

#### Composite Manufacturing

**Figure 2:**
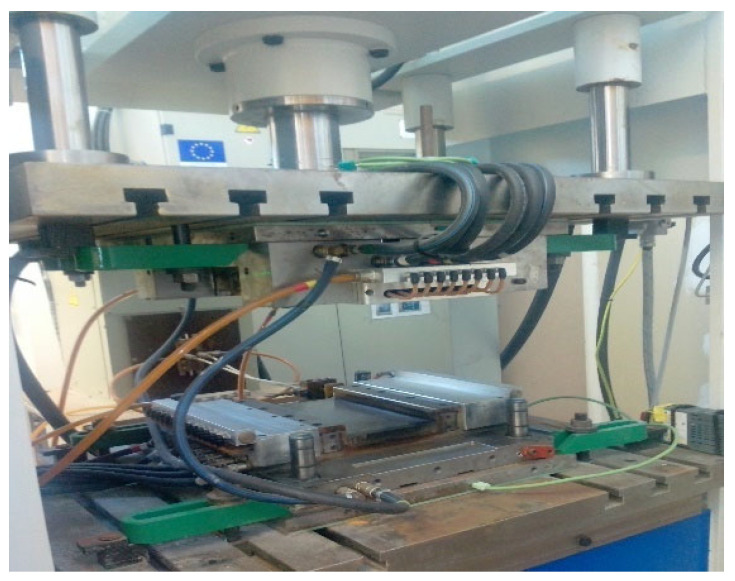
Compression molding device.

PA6-resin-based composites reinforced with Kevlar and basalt textiles were designed and manufactured utilizing a compression molding process. It is one of the low-cost molding methods as compared to other methods. By applying pressure, the compression molding process may produce significantly compact components in short manufacturing runs. As thermoplastic resin is solid at room temperature, it is somehow difficult to impregnate the reinforcement fabrics. The resin must be heated to the melting point; pressure is required to impregnate the fibers; the composite must be cooled under this pressure. Before impregnation, the PA6 resin was heated at 80 °C for 60 min in an oven (ETUVES, Chelles, France). Its melting temperature is 220 °C and degradation starts at 300 °C. Polyamide 6 exhibits superior thermomechanical properties, as well as high creep and fatigue resistance. Thermoplastic resin has an edge over its thermoset counterparts because the resin can be recovered by dissolution. The fast induction-heated compression molding device Kompass (Praha, Czech Republic), as shown in Figure 2, was used to produce lightweight thermoplastic components. The samples were placed in the compression molding machine by using a releasing agent (ZYVAX semi-permanent multiple releasing agent) on the plate. For the development of samples, the temperature was maintained at 250 °C for 4 min for proper infusion of the matrix. A 40-bar pressure was applied to produce compact composites. Metallic molds with dimensions of 30 cm × 30 cm × 3 mm were used. Composite samples of 30 cm × 30 cm size were developed. Samples were weighed before and after impregnation to determine the fiber volume fraction (Vf).

### 2.3. Characterization

#### 2.3.1. Mechanical Properties

The tensile strength and modulus of all composite samples were determined according to standard procedure of ASTM D3039 on the Zwick/Roell (Ulm, Germany) universal tensile tester. The testing device works on the principle of a constant rate of elongation (CRE), which was set to 2 mm/min. A vernier caliper was used before testing to measure the thickness of each specimen. Tensile strength gives the in-plane mechanical behavior of the composite materials. A thin flat strip of sample having a constant rectangular cross-section, i.e., 20 cm × 2.5 cm dimension, was mounted in the grips of the universal tensile tester and monotonically loaded in tension while recording load. The effective gauge length of test samples was set as 10 cm. The maximum load of the specimens was noted before fracture or failure. By monitoring the strain and load of the specimens, the stress–strain response was plotted. From this plot, the tensile modulus and ultimate tensile stress were calculated. For each sample, 20 measurements were carried out. The mean and standard deviation were calculated.

**Figure 3:**
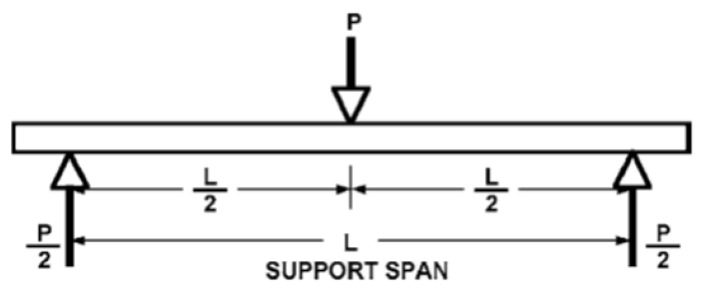
Three-point bending principle.

The flexural properties of composite samples were evaluated using the 3-point bending test according to standard test method ASTM-D7264. All tests were carried out with a span-to-thickness ratio of 32:1 and a crosshead speed of 2 mm/min. The force was continuously applied on the specimen until it fractured, or the value of force reduced to 40% of the maximum force. The Zwick/Roell (Ulm, Germany) universal testing device was used for this purpose by changing the clamps. It measures the flexural stiffness/strength of polymer matrix composites. A specimen of rectangular shape having dimensions 120 mm × 13 mm was supported at the ends and deflected at the center point. As force was applied on the specimen, it started deflecting from center. The deflection and force were measured and recorded until the failure occurred or the maximum force reduced to 40%. For each sample, 20 measurements were carried out. The mean and standard deviation were calculated. The principle of 3-point bending is shown in Figure 3. 

The gauge length/support span of 80 mm, deformation rate of 1 mm/min, and load of 5 kN were maintained. The flexural strength was calculated using Equation (1).

(1) $$\sigma \;=3\mathrm{PL}/2{\mathrm{bh}}^{2}$$

The flexural modulus was calculated using Equation (2).

(2) $$E\;={\;\mathrm{PL}}^{3}/4{\mathrm{ybh}}^{3}$$

where P represents the load, L represents the gauge length, b represents the width, h represents the thickness, and y represents the deflection during bending. 

In order to investigate the impact properties of the samples, a Zwick/Roell HIT 50P Charpy impact tester (Ulm, Germany) was used according to the ISO-179-1 standard. Samples were cut in a size of 80 mm × 10 mm for testing. The thickness and width of samples were measured by a vernier caliper before testing. Specimens were notched on one side for initiation of a predetermined crack. They were placed on a specific slot and a pendulum with 50 J of energy was allowed to hit and break the specimens. 

The absorbed energy was recorded, and the impact strength was calculated by Equation (3).

(3) $$\partial \mathrm{cu}\;=\;\mathrm{WB}\;/\mathrm{bh}\;\times \;10^{3}$$

where

WB is the energy at break in joules;

b is the width in millimeters;

h is thickness of the specimen in millimeters.

For each sample, 20 measurements were carried out. The mean and standard deviation were calculated. The specimen testing was performed under controlled environmental conditions of 25 ± 1 °C and 65% relative humidity.

#### 2.3.2. Thermogravimetric Properties

Thermogravimetric analysis (TGA) was performed on a Mettler Toledo TGA/SDTA851e instrument (Columbus, OH, USA) to study the thermal gravimetric behavior (thermal stability and degradation) of the composite samples. It was performed under a dynamic nitrogen atmosphere. The samples were heated from room temperature to 700 °C at a heating rate of 10 °C/min to yield the decomposition temperature, mass loss, and maximum decomposition.

#### 2.3.3. Differential Scanning Calorimetry

A Differential Scanning Calorimeter DSC6 (Perkin Elmer, Waltham, MA, USA) was used to monitor the temperature and heat flow resulting from different transitions as a function of time and temperature. The samples were heated at a constant rate of 15 °C/min between temperature ranges of 25 °C to 400 °C and then cooled in nitrogen atmosphere with a flow rate of 20 mL/min.

#### 2.3.4. Morphological Analysis

The fractured surface morphology of composites after tensile testing was examined by a Navitar Macroscope (Rochester, NY, USA) with a CCD camera, imaging source, and software NIS –Elements (Melville, NY, USA). Samples were examined with appropriate magnification for obtaining high-resolution images. The interfacial bonding/fiber–matrix interaction, fracture behavior, and fiber pull-out of the samples after mechanical tests were studied from these images. Scanning electron microscopy (SEM) was carried out for the composites after mechanical (tensile) testing. The samples for the scanning electron microscope were prepared with a Quorum Q150R ES (Brno, Czech Republic), which is a sputter and uses gold-plating with an argon gas atmosphere. The thickness of the gold plating was maintained at 2 nm using a current of 20 mA. The scanning electron microscope MIRA 3 TESCAN (Brno, Czech Republic) was used for this purpose. The samples were visualized in a nitrogen atmosphere with an SE (secondary electron) detector, using an acceleration voltage of 10 kV. The working distance was maintained at 16–32 mm with the scan mode. The 100× magnification was used for all the samples.

#### 2.3.5. Statistical Analysis

ANOVA was used to study the significance of the mechanical properties of composites. A *p*-value less than 0.05 indicates statistical significance of the data. R-square indicates the effectiveness of the correlation. Minitab 21.1.0 (Philadelphia, PA, USA) was used to obtain the interval plots, which were also studied to analyze the range of variations.

## 3. Results and Discussion

The samples with hybridization and different sequences of stacking are given in Table 4. The samples were cut both in the length (warp) and width (weft) direction. Due to the multiple layering of fabrics in alternate directions, the mechanical properties of the composites were observed to be uniform in both directions and the average was calculated. The various mechanical properties that were evaluated are also presented with limits of variation. The thickness and fiber volume fraction of the samples are given in Table 5.

**Figure 4:**
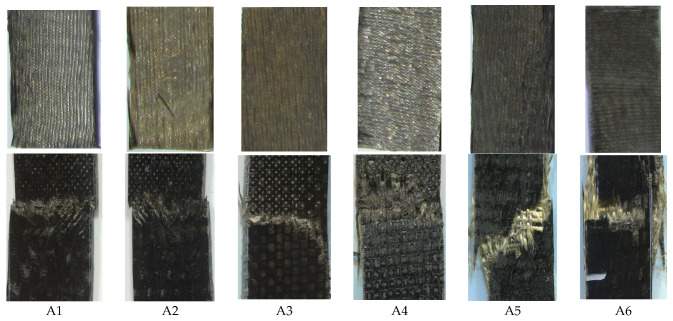
Fracture images of composite samples after tensile testing.

The composite samples and damage after tensile testing are shown in Figure 4.

From the nature of fracture, it is evident that the composites are well prepared, and the failure is catastrophic in nature. This is an indication that the load is transferred to the fabric and not the matrix. Such behavior is also a positive sign of adequate impregnation with the resin phase.

### 3.1. Characterization of Tensile Properties

**Figure 5:**
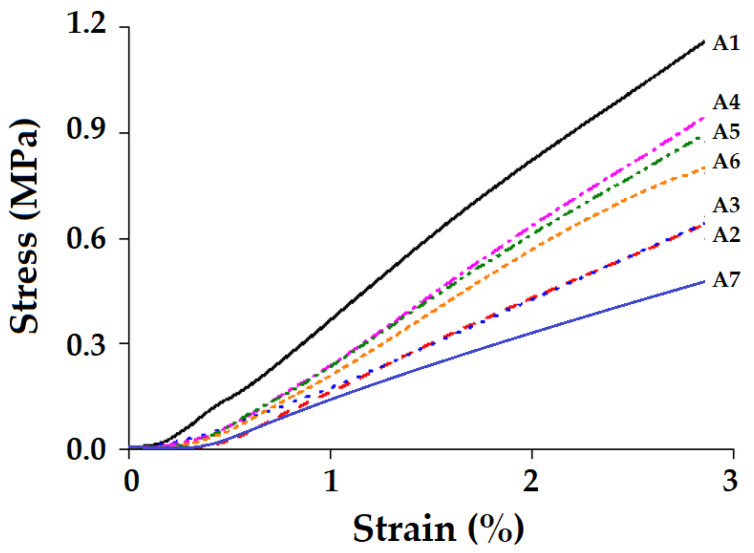
Stress-strain behavior of the composites.

The results reported in Table 4 are the average of 20 tests of tensile strength for each composite sample vis-à-vis pure resin (PA6). Mechanical properties of the composites depend on the reinforcement type. Each type of composite sample was prepared with the same thickness and weight ratio and contains four layers of reinforcement fabrics except samples A2 and A6 because a single layer of tight Kevlar has a GSM (areal density) equal to two layers of normal Kevlar. Figure 5 shows the non-linear tensile stress–strain behavior of the composites. According to the results, the A1 sample shows the maximum stress-to-strain ratio due to the high volume–mass fraction and high tensile strength of the basalt fibers [52]. A2 and A3 composites show comparatively similar results as they are made of the same fiber material. Hybrid composites (A4–A6) show improved strength and modulus as compared to A2 and A3, but show a lower strength and modulus than the A1 composite does due to the anisotropic nature of the Kevlar fabric, which does not contribute significantly in the transverse direction [53]. From the results, it can be observed that the A4 hybrid composite configuration behaves better than other hybrid combinations with respect to stress–strain behavior due to the alternative combination of basalt/Kevlar fabric in hybridization. Sample A6 shows the lowest modulus value among the hybrid samples due to the weaker adhesion with the matrix and the relatively compact structure of the tight Kevlar woven fabric, which does not allow as easy impregnation of the resin as in the case of normal Kevlar fabric.

**Figure 6:**
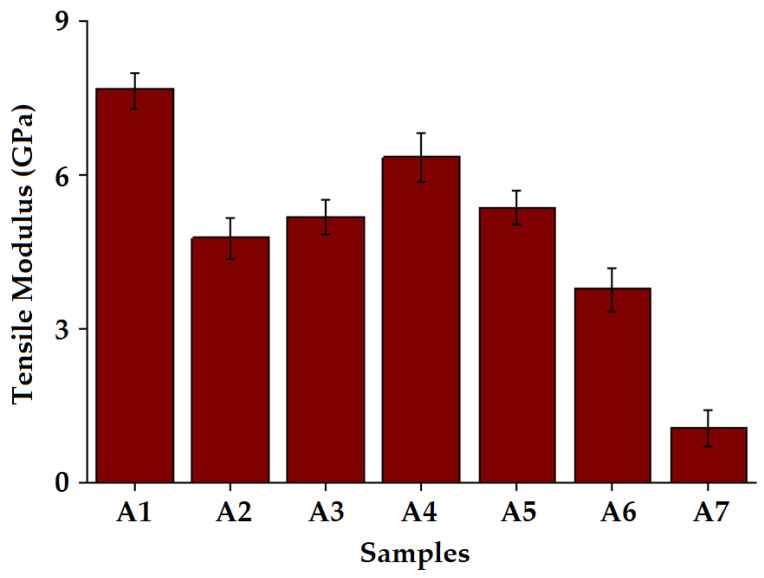
Tensile modulus of the composites.

In this study, the tensile modulus was determined by analyzing stress–strain curves. Because of the higher strength of basalt fibers, the composite sample A1 shows the highest tensile modulus values, whereas the other composites achieve lower modulus values. Among hybrid structures, the composite A4 shows superior results and follows a similar pattern as the stress/strain curve. The hybridization of Kevlar with basalt fabric results in an increase in the tensile modulus. Sample A6 shows the lowest modulus among composite samples due to the tight Kevlar structure, which might not facilitate the easy impregnation of resin. Further, the adherence of such a fabric with the adjacent layers of basalt fabrics seems to be less effective. The results are shown in Figure 6.

### 3.2. Characterization of Flexural Properties

**Figure 7:**
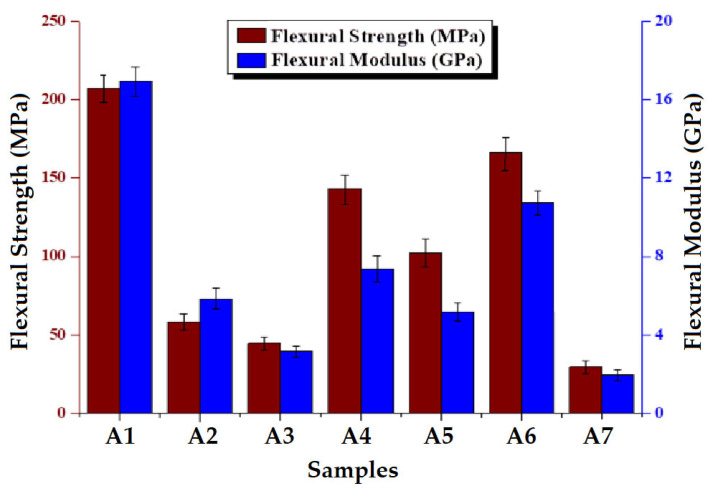
Flexural strength and modulus of the composites.

The results reported are the average of 20 tests of flexural strength for each composite sample with different combinations, mentioned in Table 1. Figure 7 shows the flexural strength and flexural modulus of the composites. Composite sample A1 shows the maximum flexural strength as well as modulus due to the higher tensile strength than others. On the other hand, pure Kevlar-based composites A2 and A3 are much weaker in flexural properties due to their lower tensile strength [54]. Hybrid composites (A4–A6) show improved flexural strength due to the supportiveness of the basalt layer. Among the hybrid composites, sample A6 shows the maximum flexural strength due to the high volume–mass ratio of the Kevlar in (tight Kevlar). The flexural modulus follows a similar trend as flexural strength.

### 3.3. Characterization of Impact Properties

**Figure 8:**
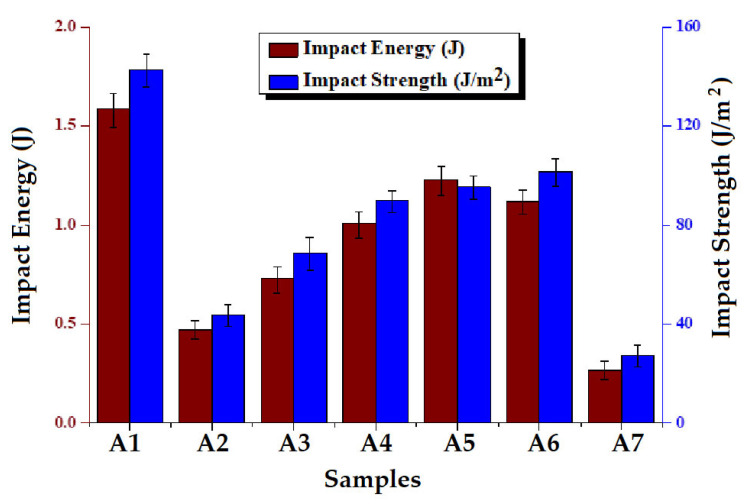
Impact energy and impact strength of the composites.

The average of 20 impact measurements is reported for each type of composite sample. All the samples show partial breakage. Figure 8 shows the impact energy absorbance capabilities of the composites. From the figure, it is clear that sample A1 shows the maximum impact strength and impact energy absorption among all the composites due to its high impact resistance [55]. Composite samples A2 and A3 show the minimum energy absorbance capabilities. Hybrid composites A4–A6 show improved energy absorbance capacity. Among hybrid composites, sample A5 shows better impact results due to the energy absorption capacity of double layers of loose Kevlar fabric in the middle layer. In sample A4, the alternate combination of basalt/Kevlar, and in sample A6, the tight Kevlar in the middle result in lower impact energy absorption capabilities. The impact strength shows the same behavior as impact energy absorbance capabilities. The higher tensile and bending strengths of basalt fabric help achieve improved impact performance in the hybrid composites. However, an appropriate layering arrangement is to be selected. Furthermore, the compression and resilience of the individual layers play an important role in determining the impact energy absorption capacities of the reinforced composites. This explains the slightly different trend in the case of impact strength and impact energy absorption [56].

### 3.4. Statistical Analysis of Mechanical Properties

One-way analysis of variance (ANOVA) was carried out in order to study the significance of the results obtained through mechanical testing in the tensile, flexural, and impact category. The results of *p*-value and R-square are given in Table 6.

**Figure 9:**
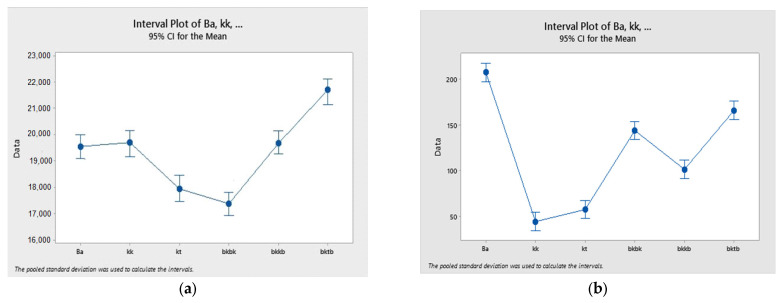

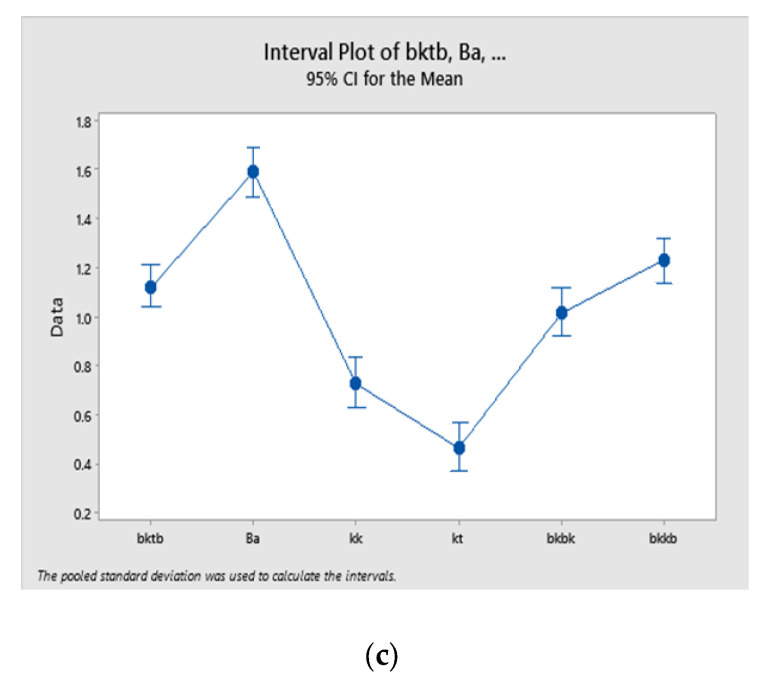
Interval plot to study the effect of fiber and their hybridization on mechanical properties: (**a**) Tensile properties, (**b**) Flexural properties, and (**c**) Impact properties of composites.

From Table 6, it is evident that the *p*-value of all responses are less than 0.05, which indicates a significant effect of fiber properties on the responses (mechanical properties of composites). The R-square value obtained for tensile properties is lower, which means that the type of fiber is less effective in determining the tensile properties among the samples studied. The interval plots are shown in Figure 9. 

Interval plots of all properties were also studied. The overlapping of samples in this plot shows an insignificant difference between their mean values. Especially in case of tensile properties, the overlapping indicates that the difference in means is not as significant as in the case of flexural and impact properties.

### 3.5. Morphological Analysis of Fracture Surfaces

**Figure 10:**
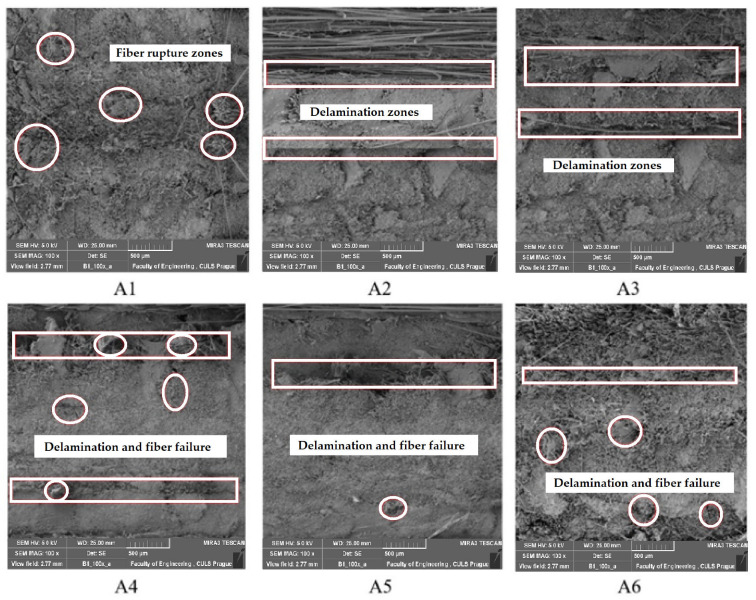
SEM images of fractured composite samples.

The composite samples after tensile failure were analyzed. SEM images were taken to study the fiber rupture, pullout, or delamination between layers of fabrics in the composites. The images are shown in Figure 10.

From the SEM images, it is visible that sample A1 mostly shows fiber rupture. This is indicative of excellent consolidation among the different layers of the basalt fabric. Thus, the sample exhibited the best tensile, bending, and impact performance. Samples A2 and A3 mostly show delamination zones, indicating poor bonding between the layers of Kevlar fabric. In the case of hybrid samples (A4–A6), there are some delamination and some fiber rupture zones. This indicates that the load is distributed between the fabric layers as well as among the fibers in each layer. Thus, an improved mechanical performance is observed as compared to pure Kevlar-fabric-based composites. However, the optimum load distribution depends on the appropriate selection of layer sequencing and the density of individual layers of component fabrics.

### 3.6. Thermogravimetric Analysis

**Figure 11:**
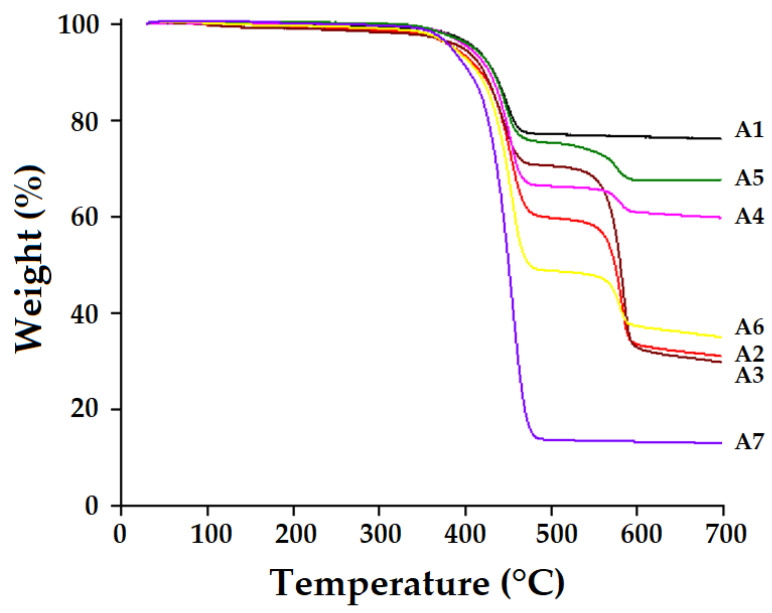
TGA curves of the composites and pure resin (PA6).

Thermogravimetric analysis was carried out in order to determine the thermal stability of composite samples across the temperature limits they may be exposed to before they begin to show signs of wear and tear. TGA was used to determine the weight loss of the composite samples as a function of increasing temperature. To assess the thermal stability of composites, the TGA study was carried out, which was followed by comparative analysis between various kinds of composites, as shown in Figure 11. 

The thermogram shows a progressive weight loss as the temperature is raised, with the weight loss beginning in the interval of 360–390 °C. It has been discovered that quantitative chain rupture causes a significant deterioration phase in neat polyamide-6 resin at temperatures between 360 and 400 °C. Other studies have also reported that the maximum degradation for pure polyamide-6 occurs at this temperature [57]. Under extreme temperatures, the composites degrade in two stages: first, they become brittle and then crumble [58]. The temperature ranging from 360 to 390 °C denotes the first stage, which corresponds to the decomposition of the matrix. The temperature ranging from 500 to 580 °C indicates the second stage, which corresponds to the decomposition of fabrics and the decomposition temperature of the Kevlar fiber. Sample A1 exhibits the highest resistance against thermal degradation with the least amount of weight loss. Following that, hybrid structures such as A5, A4, and A6 result in reasonably improved resistance to degradation and lower weight loss. Because Kevlar has a lower degradation temperature as compared to basalt, it could not perform as well as the basalt sample. This was observed when Kevlar is used as an exterior layer in hybrid samples and also as a pure component in the composites. Sample A4 results in a higher weight loss as compared to A3 owing to the use of Kevlar on the outer layers of the composite on one side of the composite. However, as compared to other hybrid constructions, the composite sample A6 has one thick layer of dense Kevlar. That is why it results in a higher weight loss and lower resistance to thermal degradation. Sample A2, which is composed of tight Kevlar, achieves slightly better outcomes than the composite sample A3, which is composed of loose Kevlar. The pure matrix/resin sample A7 degrades linearly in a single step. The temperature range of 360–398 °C denotes the decomposition of PA6 resin. For the pure basalt sample, there is a minimal loss of weight until 700 °C, which is mainly due to decomposition of the matrix/resin phase. The basalt fiber is highly resistant to thermal degradation and almost remains intact [59]. The use of this material in pure or hybrid form improves the overall thermal stability of the composite. This observation is supported by the findings of the FTIR analysis (ATR mode) and EDX of the basalt fabric. Basalt is mainly composed of Si, Al, and C, which are responsible for the thermal stability of the overall composite. 

### 3.7. Differential Scanning Calorimetry (DSC) Analysis

**Figure 12:**
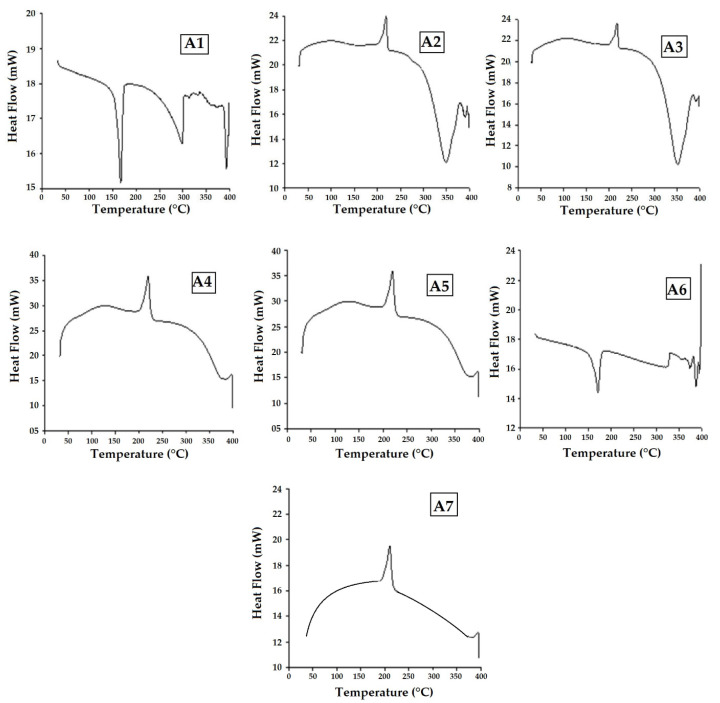
DSC analysis of the composites and pure resin (PA6).

The differential scanning calorimeter (DSC) monitors the heat flow resulting from different transitions as a function of time and temperature. This approach provides both qualitative and quantitative information on physical and chemical changes that occur because of endothermic (heat absorption) and exothermic (heat release) processes, respectively. The magnitudes of the exothermic and endothermic peaks reflect the thermal phase change of the composites [60,61,62]. The glass transition temperature (Tg) of the matrix in the composites is critical as it determines how the materials behave at various temperatures. Below this temperature, the materials become stiffer, and only little deformation occurs when the materials are subjected to thermal loading. When the temperature is raised beyond this point, the material shows rubber-like properties [62]. Figure 12 shows the DSC curves of the composites that were studied, in which two events are more prevalent than the others (endothermic and exothermic). 

It is observed that the endothermic peak emerges in composite samples A1 and A6 as the temperature increases from room temperature to 180 °C. It is linked to the dehydration process of the composite samples. However, the endothermic peak of the other composite samples A2–A5 is somewhat greater, reaching a temperature around 350–370 °C, owing to the hybridization effect of sandwich materials. As is well known, the greater the adhesion, the higher the energy needed to break the bonds. A possible explanation is that composites with a hybrid effect of basalt and Kevlar fibers exhibit dehydration and bond breakages at much higher temperatures due to the adhesivity of the adjacent layers. It is observed that the pure-Kevlar-fiber-based samples A2 and A3 do not show the endothermic peak. This may be attributed to the higher crystallinity and lower moisture content of the fibers as compared to basalt. The exothermic peak in samples A2–A5 is seen at about 200–220 °C, which is caused by the degradation of the matrix (PA6). The hybridization of basalt and Kevlar offers higher peaks of heat flow. This indicates better thermal stability of such composite materials. As compared to the pure resin, the composite samples show significantly superior thermal peaks and, therefore, superior thermal stability.

A summary of the composites developed in terms of their practical application is shown in Table 7.

## 4. Conclusions

The basalt- and Kevlar-fiber-reinforced hybrid thermoplastic polymer composites with different stacking sequences were fabricated and evaluated. The mechanical and thermomechanical properties were investigated. According to the results of the mechanical testing of the composites, the pure basalt composite exhibited the highest tensile strength and modulus. Pure-Kevlar-based samples produced results that were comparable to one another but inferior to pure basalt. In comparison to the pure-Kevlar-based composite, hybrid composites demonstrated a higher strength and yielded a significantly higher modulus. In the stress–strain curve, the A4 hybrid composite structure performed much better than the other hybrid configurations did, due to the most adequate layering sequence and hybridization. Pure-basalt-based composites exhibited a significantly higher flexural strength and modulus as compared to pure Kevlar-based composites. Hybrid composites showed better flexural characteristics because of the basalt fibers. Composite sample A6 exhibited the highest flexural strength among the hybrid composites due to the dense layer of Kevlar in the core. The highest impact strength was observed for the pure-basalt-fabric-based composite. The hybrid composites exhibited a higher energy absorption capability as compared to the pure Kevlar sample. Sample A5 performed better than other hybrid composites due to the presence of two layers of normal Kevlar in the core of the composite.

The TGA analysis showed a gradual weight loss as the temperature was elevated, with the weight loss commencing at around 360–390 °C. The pure-basalt-based composite achieved the best performance with minimal weight reduction. Hybrid composites performed better than pure-Kevlar-based samples due to the higher thermal stability of the basalt component. The DSC analysis also proved that the hybrid composites of basalt and Kevlar can endure higher temperatures and exhibited exothermic peaks at about 200–220 °C owing to the presence of Kevlar component.

Overall, the mechanical and thermomechanical characteristics of the basalt–Kevlar hybrid thermoplastic polymeric composites are promising, and the results of the research demonstrate that the hybrid composites can be used for high-load-bearing and high-temperature applications, e.g., defense, aerospace, automotive, and energy sectors. Due to the advanced mechanical and thermal performance of basalt/Kevlar hybrid composites, they are potential high-performance materials to be used in several other industrial practices. Further research should be conducted to understand the novel material from specific applications. Further, the durability can be studied under severe environmental conditions, e.g., in marine applications. Fatigue tests can be performed under cyclic and dynamic loading. Basalt fibers can be used in other technologies, e.g., pultrusion, winding, resin film infusion, and 3D printing.

## Figures and Tables

**Table 1:** Specifications of reinforcement fabrics.

Reinforcement	Fiber Density (g/m^3^)	Fabric Thickness (mm)	Areal Density (g/m^2^)	End/cm	Picks/cm	Image
Basalt fabric	1.44	0.42	363	6	4	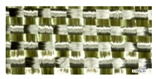
Standard Kevlar fabric	2.70	0.52	207	6	6	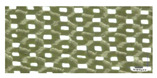
Dense/tight woven Kevlar fabric	2.70	0.69	424	18	7	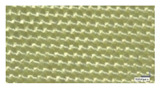

**Table 2:** Characteristics of high-fluidity Polyamide (PA6) at 65% RH (Solvay Chemicals, Brussels, Belgium).

Polyamide (PA6) Resin	
Viscosity at 285 °C (Pa.s)	30
Melting temperature Tm (°C)	220
Curing Temperature Tc (°C)	184
Elastic modulus E (GPa)	2.8

**Table 3:** Elemental composition of basalt.

Element	Weight %	Chemical Composition	Weight %
C	12.37		
O	32.98		
Na	1.64	Na_2_O	3.34
Mg	2.16	MgO	4.63
Al	8.69	Al_2_O_3_	17.50
Si	24.58	SiO_2_	52.80
K	1.34	K_2_O	1.46
Ca	6.03	CaO	8.59
Fe	9.40	Fe_2_O_3_	10.30
Ti	0.81	Others	1.38
Total:	100.00	Total:	100.00

**Table 4:** Stacking sequence of fabrics in the composite samples and their mechanical properties.

Stacking Sequence of Fabric in the Composite	Code	Maximum Force ± 0.05 (N)	Tensile Strength ± 0.05 (MPa)	Tensile Modulus ± 0.05 (GPa)	Bending Strength ± 0.05 (MPa)	Bending Modulus ± 0.05 (GPa)	Impact Energy ± 0.05 (J)	Impact Strength ± 0.05 (J/m^2^)
Basalt (B)	A1	6871.35	1.15	7.68	208.01	14.80	1.59	142.88
Tight Kevlar (KT)	A2	6916.54	0.59	4.77	55.17	5.11	0.47	43.45
Normal Kevlar (K)	A3	6225.75	0.60	5.18	42.28	2.68	0.73	68.81
Basalt/Kevlar/Basalt/Kevlar (BKBK)	A4	6152.28	0.88	6.34	94.19	3.80	1.01	89.92
Basalt/Kevlar/Kevlar/Basalt (BKKB)	A5	5503.81	0.75	5.34	130.90	5.67	1.23	95.37
Basalt/Tight Kevlar/Basalt (BKTB)	A6	4856.24	0.71	3.78	155.01	8.98	1.12	101.59
Pure Resin (PA6)	A7	2014.24	0.36	1.01	24.21	1.98	0.25	27.45

**Table 5:** Sample thickness and fiber volume fraction.

Sample Code	Thickness (mm) ± 0.01	Fiber Volume Fraction ± 0.02
A1	3.02	0.44
A2	3.01	0.45
A3	3.02	0.44
A4	3.00	0.44
A5	3.02	0.45
A6	3.01	0.46
A7	3.01	0.00

**Table 6:** One-way ANOVA of mechanical properties.

Response/Property	*p*-Value	R-Square
Tensile properties	0.001	73.14%
Flexural properties	0.004	98.06%
Impact properties	0.002	93.54%

**Table 7:** Summary of the mechanical and thermal properties of composite samples in terms of practical applications.

Stacking Sequence of Reinforcement Fabric	Code	Applications Demanding High Tensile Performance	Applications Demanding High Bending Stiffness	Applications Demanding High Impact Performance	Applications Demanding High Thermal Stability
Basalt (B)	A1	Yes	Yes	Yes	Yes
Tight Kevlar (KT)	A2	No	No	No	No
Normal Kevlar (K)	A3	No	No	No	No
Basalt/Kevlar/Basalt/Kevlar (BKBK)	A4	Yes	No	No	Yes
Basalt/Kevlar/Kevlar/Basalt (BKKB)	A5	No	No	Yes	Yes
Basalt/Tight Kevlar/Basalt (BKTB)	A6	No	Yes	Yes	No
Pure Resin (PA6)	A7	No	No	No	No

## Data Availability

Data will be made available on request.
